# Complex structural dynamics of nanocatalysts revealed in *Operando* conditions by correlated imaging and spectroscopy probes

**DOI:** 10.1038/ncomms8583

**Published:** 2015-06-29

**Authors:** Y. Li, D. Zakharov, S. Zhao, R. Tappero, U. Jung, A. Elsen, Ph. Baumann, R.G. Nuzzo, E.A. Stach, A.I. Frenkel

**Affiliations:** 1 Department of Physics, Yeshiva University, New York, New York 10016, USA; 2 Center for Functional Nanomaterials, Brookhaven National Laboratory, Upton, New York 11973, USA; 3 Department of Chemistry, University of Illinois at Urbana-Champaign, Urbana, Illinois 61801, USA; 4 Photon Sciences Division, Brookhaven National Laboratory, Upton, New York 11973, USA; 5 School of Chemical Science and Engineering, KTH Royal Institute of Technology, Stockholm 10044, Sweden

## Abstract

Understanding how heterogeneous catalysts change size, shape and structure during chemical reactions is limited by the paucity of methods for studying catalytic ensembles in working state, that is, in *operando* conditions. Here by a correlated use of synchrotron X-ray absorption spectroscopy and scanning transmission electron microscopy in *operando* conditions, we quantitatively describe the complex structural dynamics of supported Pt catalysts exhibited during an exemplary catalytic reaction—ethylene hydrogenation. This work exploits a microfabricated catalytic reactor compatible with both probes. The results demonstrate dynamic transformations of the ensemble of Pt clusters that spans a broad size range throughout changing reaction conditions. This method is generalizable to quantitative *operando* studies of complex systems using a wide variety of X-ray and electron-based experimental probes.

## Abstract (web summary)

Studying a catalyst during reaction (*operando* conditions) can give significant insights into the changes a catalyst undergoes. Here, the authors use an *operando* approach to correlate X-ray spectroscopy and electron based imaging techniques to measure the dynamic changes in Pt nanoparticles during the catalytic hydrogenation of ethylene.

## Graphical abstract

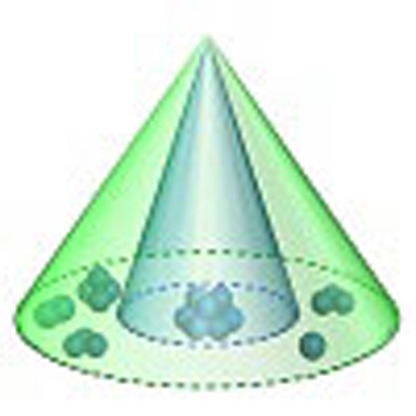

While it is well established that the size, shape, composition and atomic structure of supported metal catalysts impact reactivity, quantitative understandings of these attributes and the environmental influences that modify them in *operando* conditions remain limited. This reflects limitations due to the paucity of *operando* methods that can assess atomistic nature of composition and bonding present in complex heterogeneous systems. Here we quantitatively describe the complex structural dynamics exhibited by an exemplary catalytic reaction—ethylene hydrogenation carried out over supported Pt catalysts. The current work exploits a microfabricated catalytic reactor designed for correlated use with both synchrotron X-ray absorption spectroscopy and scanning transmission electron microscopy. The results demonstrate dynamic transformations of the ensemble of Pt clusters that spans from single atoms to large agglomerates throughout changing reaction conditions. This method is generalizable to quantitative *operando* studies of complex systems using a wide variety of X-ray and electron-based experimental probes.

Supported metal nanoparticles are commonly used in heterogeneous catalysis, and as such are critically important to myriad industrial processes^1^. They generally possess a large variety of three-dimensional structures, which are known to directly affect their catalytic function^2^. Features such as cluster size, state of atomic order, bond strain, facet orientation, the support, and bi- (multi) metallic composition have all been shown to affect catalytic activity, selectivity and stability^3^,^4^,^5^. These heterogeneous materials are exceptionally complex from a structural perspective and, even for cases of carefully prepared model systems, may demonstrate a coexistence of different particle sizes, shapes, compositions and degrees of crystalline order within the metal clusters of the catalyst^6^,^7^,^8^. This heterogeneity poses a formidable challenge to ensemble-averaging characterization techniques such as X-ray absorption fine structure (XAFS) spectroscopy, which collapses this structural heterogeneity into a single average measurement, albeit with exquisite spatial resolution^9^. Techniques such as electron microscopy can be used to directly measure the structural heterogeneity in a sample and atomic displacements within individual nanoparticles with sub-pm precision^10^, but can suffer from limited statistics and experimental artefacts associated with beam damage, insufficient resolution and/or lack of signal to noise. Thus, it is increasingly common to utilize a combination of methods to accurately describe the structural complexities that are inherent in these nanoscale systems^11^,^12^.

Further complications arise when considering the evolution of supported nanoparticles subjected to reaction conditions. It has become increasingly apparent that they can exhibit substantial structural changes when they are in a working state^13^,^14^,^15^,^16^ and that these changes may no longer be apparent when the sample is returned to a non-working state or examined *ex situ*^16^,^17^. A variety of characterization techniques have been developed in recent years that allow these structural changes to be either inferred or directly observed *in situ* (that is, when subjected to approximate working conditions through the application of temperature and pressure) or under *operando* conditions (that is, during the catalytic reaction itself, with the products being detected simultaneously during the characterization)^6^,^7^,^18^,^19^.

In this study, we have investigated the structural and electronic transformations that occur across different length scales in different components of a commonly used catalyst during its active mediation of a catalytic reaction. The work specifically investigates the active structures of silica-supported Pt clusters mediating the process of ethylene hydrogenation. Previous studies by *operando* XAFS have uncovered irreversible structural transformations in this system when it is cycled between hydrogen- and ethylene-rich environments^20^. *Ex situ* scanning transmission electron microscopy (STEM) studies showed that the average particle size is approximately the same after the reaction, but the measured variation was much smaller than the precipitous drop in the metal-metal coordination number measured by *operando* extended XAFS (EXAFS)^20^. Several scenarios could be responsible for this discrepancy, among them formation of carbidic speciations of the Pt^20^ and/or partial redispersion of the catalyst particles. Understandings of such gross transformations of material compositions and structure *in operando* are of critical importance given the need for new atomistically rationalized principles that can guide the design of catalyst systems offering better measures of selectivity, rate and stability. Data from XAFS alone cannot provide direct evidence in support of mechanisms of the type noted above (or, for that matter, any single mechanism) because they are inherently averaged over an ensemble comprising particles of different sizes and states of order^21^. This system is thus ideal for a correlated study using combined techniques: it clearly highlights the limitations of the ensemble-averaging method (XAFS) in that it is unable to fully describe the details of the particle size distribution, while the statistical method (STEM) that is most suitable for such analysis must generally be applied *ex situ* and *ex post facto*. In this work, we have coupled the two methods by using a portable micro-reactor compatible with both *operando* STEM and *operando* XAFS measurements. Using the online gas analysis carried out at each facility (electron microscope and synchrotron beamline) during the chemical transformations in the micro-reactor, we can directly correlate the results at each stage of the reaction.

## Results

### Experimental set-up of combining *operando* STEM and XAFS

**Figure 1:**
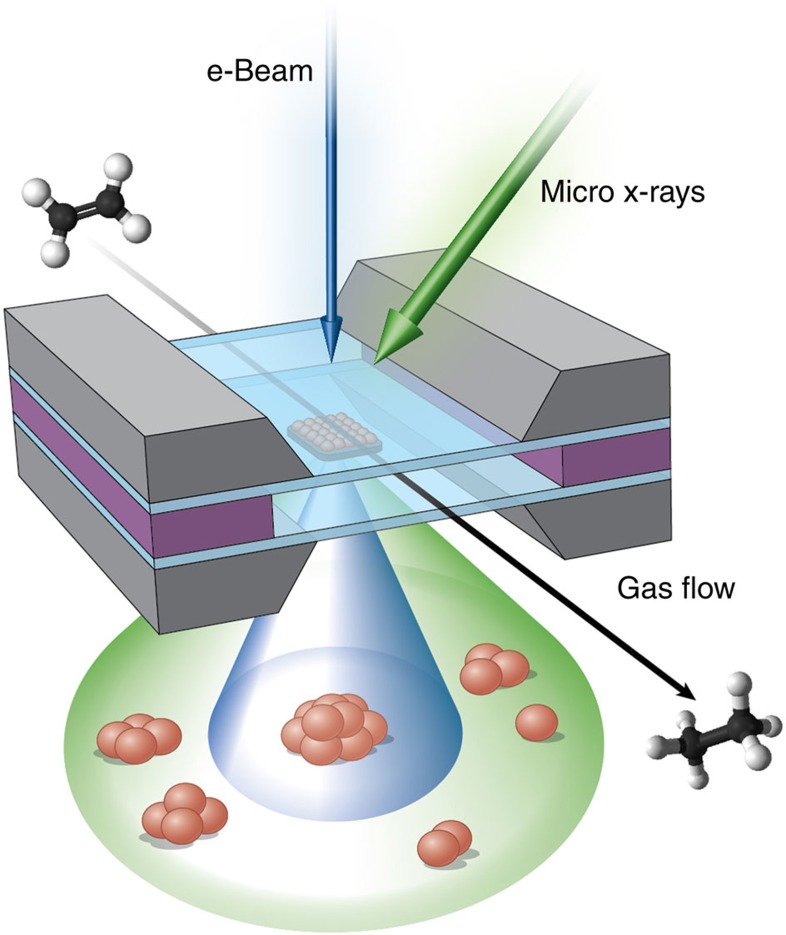
Schematic of experimental cell. The catalyst is confined between two silicon nitride windows with the reacting gas mixture flowing through the system. Arrows show the direction of the electron beam and incident X-ray beam. In the X-ray absorption experiment, all types of Pt species are probed (shown by a green cone). In the STEM experiment, only particles larger than ∼1 nm are detectable (shown by a dark blue cone).

Our experimental set-up (Fig. 1) consisted of a micro-reactor system based on silicon micromachining^22^. The catalytic nanoparticles are confined between two thin SiN ‘windows', separated by an integrated spacer. This system is integrated within a TEM sample holder so that gases can be provisioned to the sample in a sequential manner. The reaction gases were either pure hydrogen or the hydrogen and ethylene mixtures in ratios of 3:1 and 1:3 (the detailed reaction sequence is given in the Methods). X-ray studies were carried out at the micro-focusing X-ray beamline X27A at the National Synchrotron Light Source and electron microscopy characterization at the FEI Titan environmental transmission electron microscope at the Centre for Functional Nanomaterials, both at Brookhaven National Laboratory. These experiments were performed successively, first at the beamline and then at the TEM facility, using the same micro-reactor and gas input/output systems at both facilities. Residual gas analysis performed during the reactions at both facilities showed formation of ethane whenever the ethylene and hydrogen mixture was present (Supplementary Fig. 1). In both cases, ambient temperature and pressure were maintained in the cell: this is in stark contrast to conventional STEM imaging, which is normally done in high-vacuum conditions.

### Characterization of catalysts during different reaction conditions

**Figure 2:**
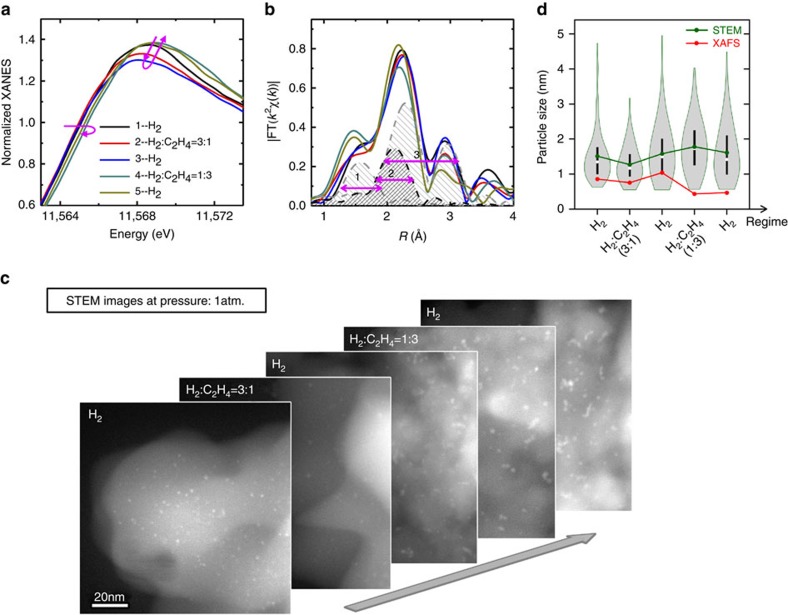
The experimental results from XAFS and STEM. (**a**) The XANES spectra, (**b**) the Fourier transform magnitudes of Pt L_3_ edge EXAFS spectra, and (**c**) the STEM images are shown as measured in the *operando* mode, during different reaction regimes. Theoretical fits to EXAFS data included three contributions between Pt and their nearest neighbours. Combined EXAFS and STEM results are displayed in (**d**) for all regimes. The green line indicates the mean particle sizes, as obtained by the STEM image analysis. The red line shows the change in the average particle size obtained from XAFS analysis, assuming that all particles are identical and adopt a truncated cuboctahedron geometry. The violin plots^23^ show more statistical information from the STEM measurements: the overall shape of the distribution is presented at each stage as the shape of the ‘violin', as are the medians (white circles) and the 25th and 75th percentiles of the data (black lines).

Figure 2a presents data from X-ray absorption near edge structure (XANES) measurements made during various stages of reaction. During the first three steps of the reaction (from H_2_ to 3:1 H_2_:C_2_H_4_ and then back to H_2_) the Pt L_3_ edge peak intensity decreases and the edge energy shifts to lower energies, indicating that the particles are being reduced. During the first two stages, no substantial changes occur in the particle structure, as illustrated by the stability of the EXAFS signal (Fig. 2b). STEM images taken in the same atmospheric pressure conditions during these same stages of the reaction yield additional details regarding the particle size distribution (Fig. 2c and Supplementary Figs 2 and 3). Most notably, following the maximum point of particle reduction (at the end of the second H_2_ exposure, indicated by the red-shift of the XANES signal) there is a marked increase in the number of agglomerated particles.

As the feed gas concentration shifts to the ethylene rich (1:3 of H_2_:C_2_H_4_) and, finally, pure H_2_ conditions, the Pt L_3_ edge peak intensity increases, accompanied by a blue-shift of the edge energy. When combined with the observed increase in Pt-nonmetal bonding and the decrease in the Pt-Pt peak intensity observed in the Fourier transform magnitude (Fig. 2b), there is an indication of both oxidative effects and a decrease in the Pt-Pt coordination number (that is, the average size of the metal regions) during the course of this stage of the reaction, in agreement with earlier work^20^. Quantitative EXAFS analysis (Supplementary Table 1 and Supplementary Fig. 4, see also Supplementary Table 2 and Supplementary Fig. 5 for results of control experiments with Clausen cell) confirms this interpretation. However—in apparent contradiction with the XAFS data—STEM indicates an overall increase in the mean particle size (Fig. 2d and Supplementary Fig. 2).

The fact that there appears to be a contradiction in the information obtained from the quantitative XAFS and STEM measurements suggests that either one or both of the techniques is not completely describing the heterogeneity present in the sample. EXAFS—an ensemble-averaging method—yields a value for the Pt-Pt coordination number that is averaged over all Pt-containing species in the sample, from individual atoms to large clusters, weighted by their mass fraction. In contrast, the STEM measurements can probe multiple particle shapes, including sintered and agglomerated particles, but they access only the size distribution beyond a ‘resolution cutoff' that varies from single atom resolution to 1 nm, depending on the instrument and experimental conditions. In our case, because of the presence of both 1 atm of gas in the reactor cell, the two 50 nm-thick confining SiN windows, and the SiO_2_ support itself, we conservatively set the STEM resolution limit to be 1 nm (see Supplementary Discussion and Supplementary Fig. 2).

### Determination of the fraction of ultra-small clusters

To reconcile the two measurements and to bridge the two lengths scales that they independently probe, we developed the following analytical formalism. The two observables—average Pt-Pt coordination number (CN) measured by EXAFS and the particle size distribution obtained by STEM—can be linked by expressing the overall coordination number (*n*) in terms of the weighted average of two coordination numbers, *n*_1_ and *n*_2_:


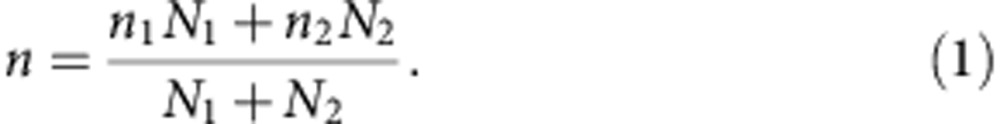


**Figure 3:**
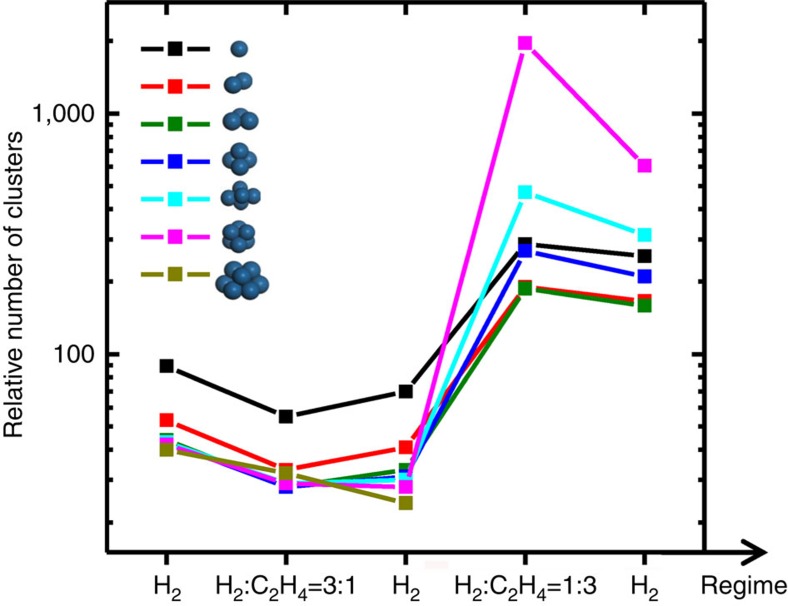
Fractions of ultra-small Pt species, sub-nm in size. Relative numbers of clusters (normalized by the number of particles counted in the STEM size distribution above the 1 nm cutoff) were obtained by combining the results of XAFS and STEM using equation 1. Numerical results are listed in Supplementary Tables 3–6.

Here *n*_1_ is the average Pt-Pt CN corresponding to the part of the particle size distribution that is below the resolution cutoff in the STEM distribution, *n*_2_ is the average Pt-Pt CN corresponding to the part of the STEM distribution that is observable, and *n* is EXAFS-obtained ensemble-average CN. The values *N*_1_ and *N*_2_ in equation 1 are the numbers of Pt atoms in the respective parts of the total distribution. Detailed procedures for calculating *n*_2_ and *N*_2_ by using the STEM-derived size distribution are described in the Supplementary Discussion. Equation 1 is identical to the principle used in balancing two objects, with masses *M*_1_ and *M*_2_ and positions *x*_1_ and *x*_2_ on a mass-less beam on opposite sides of the pivot located at position *x*. As in the case of a mechanical system designed for balancing a specific mass located in the fixed position to the right of the pivot by some variable mass and position to the left of the pivot, equation 1 does not have a unique solution for both *n*_1_ and *N*_1_. Specifically, a smaller mass located farther from the pivot will balance a given mass equally well as the larger mass located closer to the pivot. Equivalently, sub-nm Pt clusters or unreduced Pt atoms that populate the portion of the size distribution that is ‘invisible' to STEM may equally likely satisfy equation 1 when they either favour the upper end or the lower end of the 0–1 nm range, as long as the total number of atoms in such particles (*N*_1_) decreases, or increases, respectively. A characteristic that is more robust than the number of atoms is the actual number of oxidized Pt species or small Pt clusters under 1 nm in size (calculation results are given in Supplementary Tables 3–6). Indeed, as Supplementary Table 6 demonstrates, the numbers of different types of small Pt clusters calculated using equation 1 vary relatively weakly with Pt cluster size within each reaction regime. Figure 3 and Supplementary Table 6 demonstrate that the number of small clusters increases during the reaction by an order of magnitude, regardless of the exact choice of cluster size (*n*_*1*_). Importantly, it stays large after ethylene is replaced by hydrogen at the end of the process. This indicates that the change occurring in the distribution under this condition is in fact irreversible. The increased number of ultra-small Pt species during the reaction was validated by *ex situ* STEM measurements conducted before and after the reaction (Supplementary Fig. 8).

## Discussion

The following quantitative picture emerges from a holistic overview of the data, combining insights from XANES, EXAFS, STEM and reactivity data. The catalytic system is markedly heterogeneous, consisting of a population of oxidized species, ultra-small clusters (<1 nm), larger particles and agglomerates whose relative numbers,*ν*, change substantially at each stage of the reaction:


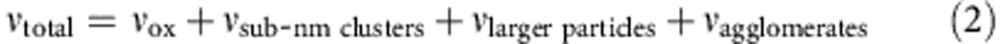


**Figure 4:**
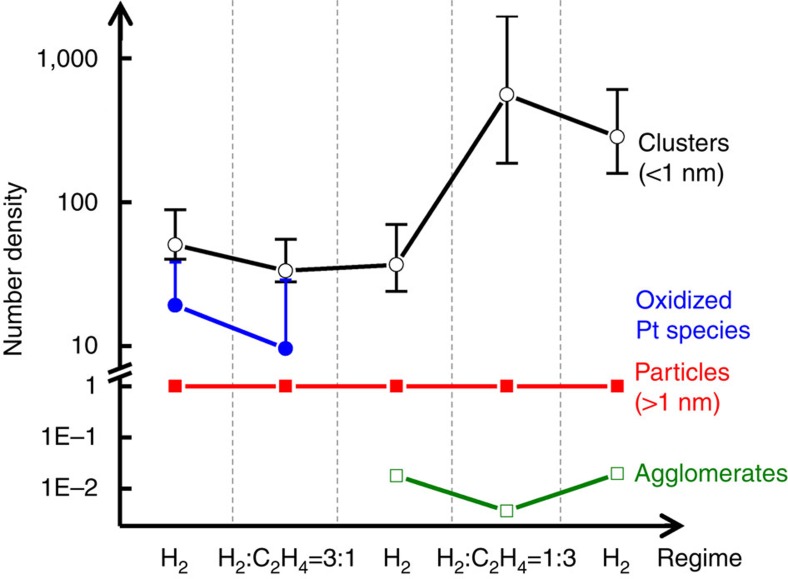
Evolution in number density of Pt species (schematic). Combination of XAFS and STEM analyses accounts for the following components and for their evolution in reaction conditions: oxidized Pt species (blue), clusters smaller than 1 nm in size (black), agglomerated clusters, or ‘agglomerates' (green) and particles larger than 1 nm in size (red).

Figure 4 gives quantitative estimates of these fractions, or number densities, normalized by the number of particles in the visible part of the size distribution, as measured by *operando* STEM in each regime (Supplementary Table 5). Figure 4 shows that at the first three stages of the process the number density of sub-nm clusters remains relatively small, compared with the last two stages, but is significantly greater (one or two orders of magnitude, depending on the stage of the reaction, see Supplementary Table 6) than the number density of larger particles observed by *operando* STEM. The number density of oxidized species gradually decreases in the first stages of the reaction, as evidenced by both the Pt L_3_ peak intensity's monotonic decrease and the decrease in the estimated number density of individual Pt atoms (Fig. 3). The formation of agglomerated particles was observed during the third stage, in pure H_2_ flow, after prior exposure to the 3:1 H_2_:C_2_H_4_ mixture. This occurred simultaneously with the completion of the reduction of the Pt (Fig. 2a). Correlating the two observations allows us to hypothesize that reduction of the oxidized Pt species that were present in the fresh sample was a necessary step that enabled agglomeration of ultra-small clusters into larger particles. Notably, the observation of dumbbell-shaped particles in our measurement (Supplementary Fig. 6 and Supplementary Table 7) is a tell-tale sign of the diffusion-coalescence mechanism of cluster growth, in which cluster fragments become mobile and combine into larger particles. An alternative model of growth, Ostwald ripening, requires the observation of a bimodal distribution of particle size that continues to show increased numbers of larger particles with time. This trend was not observed in our data under any condition examined experimentally.

The fact that the number of small clusters markedly increased in stages 4 and 5 is not related to reduction of oxidized Pt species, because the reduction is all but completed by the end of the 3rd stage, without there being a noticeable change in the number of sub-nm clusters in the first three stages (Fig. 3). In addition, the behaviour of the Pt L_3_ XANES peak (Fig. 2) undergoes a significant change in trend: during the first part of the reaction sequence the edge peak decreases monotonically from stages 1 to 3, whereas it increases from stage 3 to stages 4 and 5. Thus, the inferred, large increase in the number of sub-nm clusters at stages 4 and 5 must have a different origin from that of any species produced via the reduction mechanism. The present data suggest that the increased numbers of sub-nm clusters must be due predominantly to the observed fragmentation of the larger agglomerated particles in the ethylene-rich environment (stage 4, Fig. 4). Uzun and Gates have reported analogous observations of particle fragmentation during another ethylene hydrogenation reaction, with Ir clusters supported on zeolites fragmenting in response to the provision of ethylene and hydrogen^24^.

Our case study of supported Pt catalysts reveals substantial dynamic restructuring in reaction-driven transformations of the catalyst over a wide range of length scales, from single atom to larger clusters. The chief among them was the fragmentation of the metal clusters in the sub-nm range, preceded by reduction of oxidized metal species present in the fresh sample, and followed by the coalescence of the clusters into larger particles. Only by coupling together (via the *operando* approach) the XANES, EXAFS and STEM measurements could we adequately characterize all metal species across the range of sizes present in this exemplary supported catalyst (0–5 nm). The protocol reported here is general and can be applied to a broad class of mechanistic studies of catalytic reactions mediated by functional nanomaterials. This correlated, *operando* approach provides insights into the dynamic structural attributes of active catalytic materials over a range of characteristic sizes extending from single atoms to clusters of several nanometres in size.

## Methods

### Catalyst preparation

The SiO_2_-supported Pt catalyst was prepared by wet impregnation and subsequent reduction in hydrogen. Specifically, 7.6 mg (NH_3_)_4_Pt(OH)_2_ (Alfa) was dissolved in 3 ml of water and 500 mg SiO_2_ (Sigma, 99.5%) was added to this precursor solution, in agreement with a nominal weight loading of 1%. The resulting suspension was dried under stirring at 50–60 °C. Afterwards, the sample was heated in a tube furnace at a rate of about 10 K min^−1^ to 250 °C in Ar flow, reduced in 5% H_2_ in N_2_ at this temperature for 120 min, and then slowly cooled down. Finally, the sample was purged in Ar.

### Description of *operando* microreactor

The *operando* microreactor used was adapted from a commercially available liquid-cell transmission electron microscopy sample holder (http://hummingbirdscientific.com/products/liquid). The Pt/SiO_2_ catalysts were loaded between two 50-nm-thick SiN windows, created through microfabrication and spaced 500 nm apart at their nearest point. A custom-built gas-flow reactor was built for the holder to provide the reactant gas stream. It consisted of three mass-flow controllers, which provided He, H_2_ and C_2_H_4_. A quartz capillary (a.k.a. Clausen cell) of 0.9 mm I.D. and 1.0 mm O.D and loaded with the catalyst was located downstream of the micro-reactor, and the product analysis was done for the output of the Clausen cell to increase the product yield detected by quadrupole mass spectrometer.

### Experimental set-up for *operando* XAFS measurements

Microbeam XAFS data in this study were collected on the bending magnet beamline X27A at the National Synchrotron Light Source (NSLS), Brookhaven National Laboratory (BNL). The X-ray beam was tuned using a Si(111) channel-cut monochromator. Monochromatic X-rays were focused to a beam size of 7 × 10 μm^2^ using Rh-coated, silicon Kirkpatrick-Baez microfocusing mirrors. The incident beam intensity was monitored using an ion chamber upstream of the focusing optics. X-ray fluorescence spectra were collected using a 4-element Vortex ME4 silicon-drift detector (SII Nanotechnology) with an active area of 200 mm^2^. X-ray transmission through the sample cell was recorded using a p-type, intrinsic, n-type (PIN) photodiode: this signal was useful for initial alignment of the cell with the micro-focused beam.

Samples were placed in the micro-reactor, which was subsequently mounted in a rod at the centre of rotation of a rotational stage (*θ*), and the rotational stage was fixed to the high-precision XYZ sample scanning stage assembly. The sample cell was designed for transmission measurements and required a unique geometry for adaptation to fluorescence detection. After the beam was threaded through the aperture of the sample cell (measured by the PIN diode intensity) the cell was rotated 45° and then repositioned with the XYZ stage to maximize transmitted signal. Vortex ME4 detector was repositioned from 90° to the incident beam to 135° to the incident beam (that is, facing upstream) and aligned to the exit window of the sample cell. Fluorescence signal from the sample material itself was used to fine tune the position of the sample cell, typically this position was a few degrees more than the initial 45° rotation. For microbeam Pt L_3_-edge XAFS spectroscopy measurements, the incident beam energy was scanned across the respective L_3_-edge using 1 to 5 eV energy steps. All fluorescence signals were normalized to changes in intensity of the X-ray beam as measured by the ion chamber (*I*_0_). Data acquisition and visualization were performed using IDL-based beamline software designed by University of Chicago, CARS group.

### Experimental set-up for *operando* STEM characterization

Electron microscopy experiments were performed using a Titan 80/300 Environmental Transmission Electron Microscope at the Centre for Functional Nanomaterials, Brookhaven National Laboratory. Annular dark field scanning transmission electron microscopy images were acquired at a constant image magnification of 640,000, as it was determined that at higher levels of magnification (and thus dose rate), the presence of hydrocarbon in the gas stream leads to strong hydrocarbon deposition in the imaging region (Supplementary Fig. 7), obscuring the visibility of the nanoparticles significantly. Images were acquired once the reaction had settled to the equilibrium reaction conditions, directly correlated with the XAFS measurements, and from different (fresh) regions of the sample.

### *Ex situ* STEM characterization

The *ex situ* STEM images of Pt/SiO_2_ catalyst before and after reaction were taken using Hitachi 2700C Scanning Transmission Electron Microscope with a probe aberration-corrector, operating at 200 kV. The images and histograms of size distribution are shown in Supplementary Fig. 8.

## Additional information

**How to cite this article:** Li, Y. *et al.* Complex structural dynamics of nanocatalysts revealed in *Operando* conditions by correlated imaging and spectroscopy probes. *Nat. Commun.* 6:7583 doi: 10.1038/ncomms8583 (2015).

## Supplementary Material

Supplementary Information Supplementary Figures 1-8, Supplementary Tables 1-7, Supplementary Discussion and Supplementary References
