# Supplementary material for: Complex structural dynamics of nanocatalysts revealed in Operando conditions by correlated imaging and spectroscopy probes
Source: Nat Commun. 2015 Jun 29;6:7583. doi: 10.1038/ncomms8583 (PMC4491830; doi:10.1038/ncomms8583)
Supplement: Supplementary Information — Supplementary Figures 1-8, Supplementary Tables 1-7, Supplementary Discussion and Supplementary References [file ncomms8583-s1.pdf]

Supplementary Figures

1. Results of reactivity measurements in correlated experiments by XAFS and STEM.

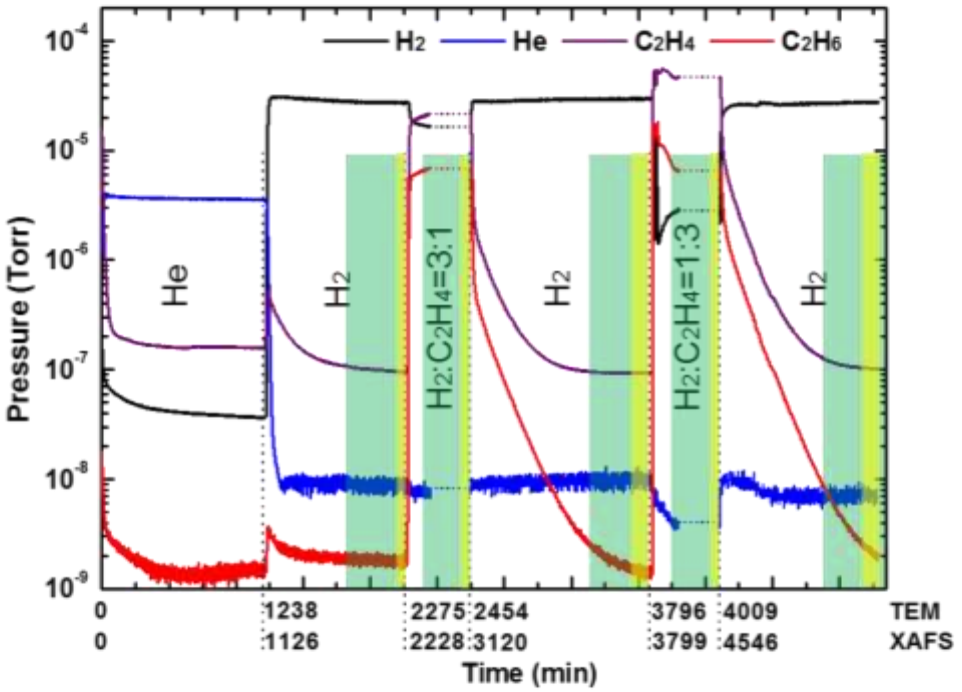

Supplementary Figure 1. The pressure of reactants and products in different regimes. The gas flow sequence is He→H_2→H_2:C_2H_4 (3:1) →H_2→ H_2:C_2H_4 (1:3)→H_2. The purpose of flowing He before introducing other gases is checking leakage of the system, initiating a clean environment for the real working condition and providing knowledge of the gas background. The green bar indicates the time period for XAFS measurements and the yellow for STEM measurements. All measurements were done when the gases reach stable state. The pressure vs. time figure is very similar in XAFS and STEM measurements, therefore only one representative figure is shown here with different time scales in x-axis for different techniques.

2. Size frequency distributions and representative images of SiO_2 supported Pt nanoparticles in different gas environments measured by *operando* STEM in a micro-reactor.

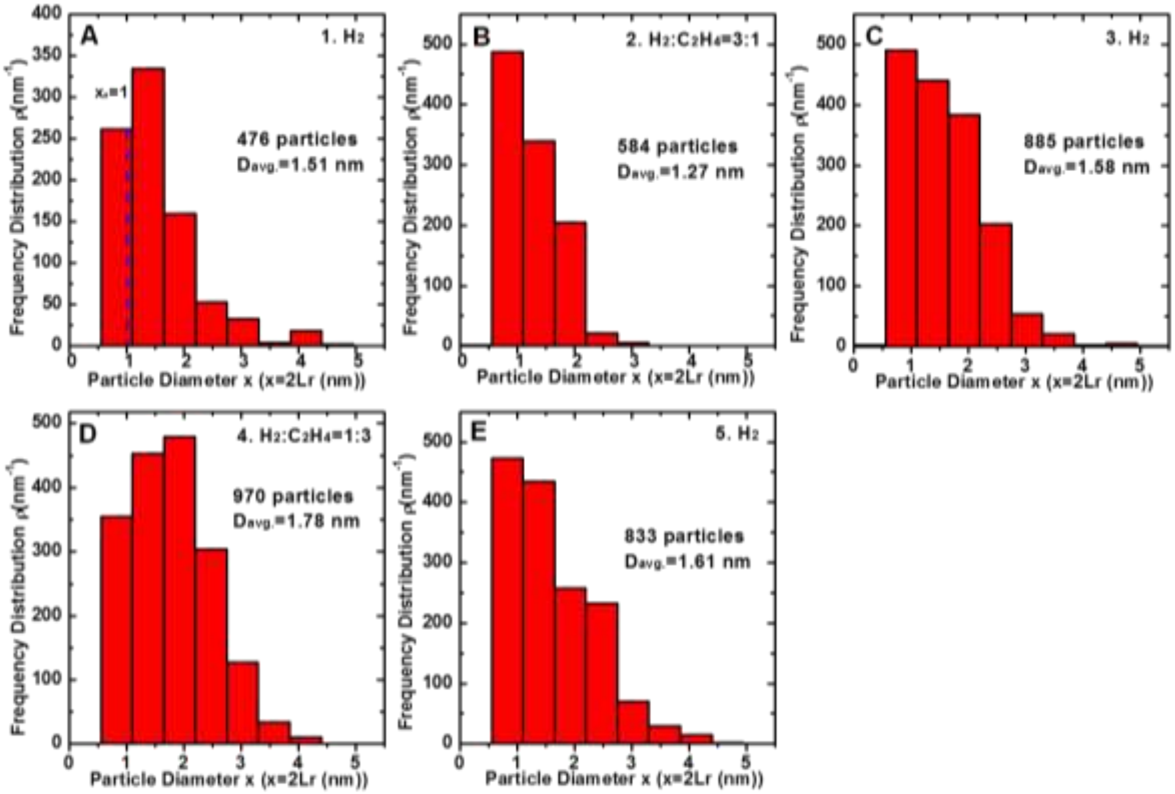

Supplementary Figure 2. Size frequency distribution of SiO_2 supported Pt nanoparticles in different gas environment. (A) initial H_2 (B) mixture of H_2 and C_2H_4 with the ratio of 3:1 (C) middle H_2 (D) mixture of H_2 and C_2H_4 with the ratio of 1:3 (E) final H_2. In our STEM experiments, the cutoff (the resolution limit) corresponds to x_a = 1nm for all distributions (the cutoff is only shown in Supplementary Figure 2(A)).

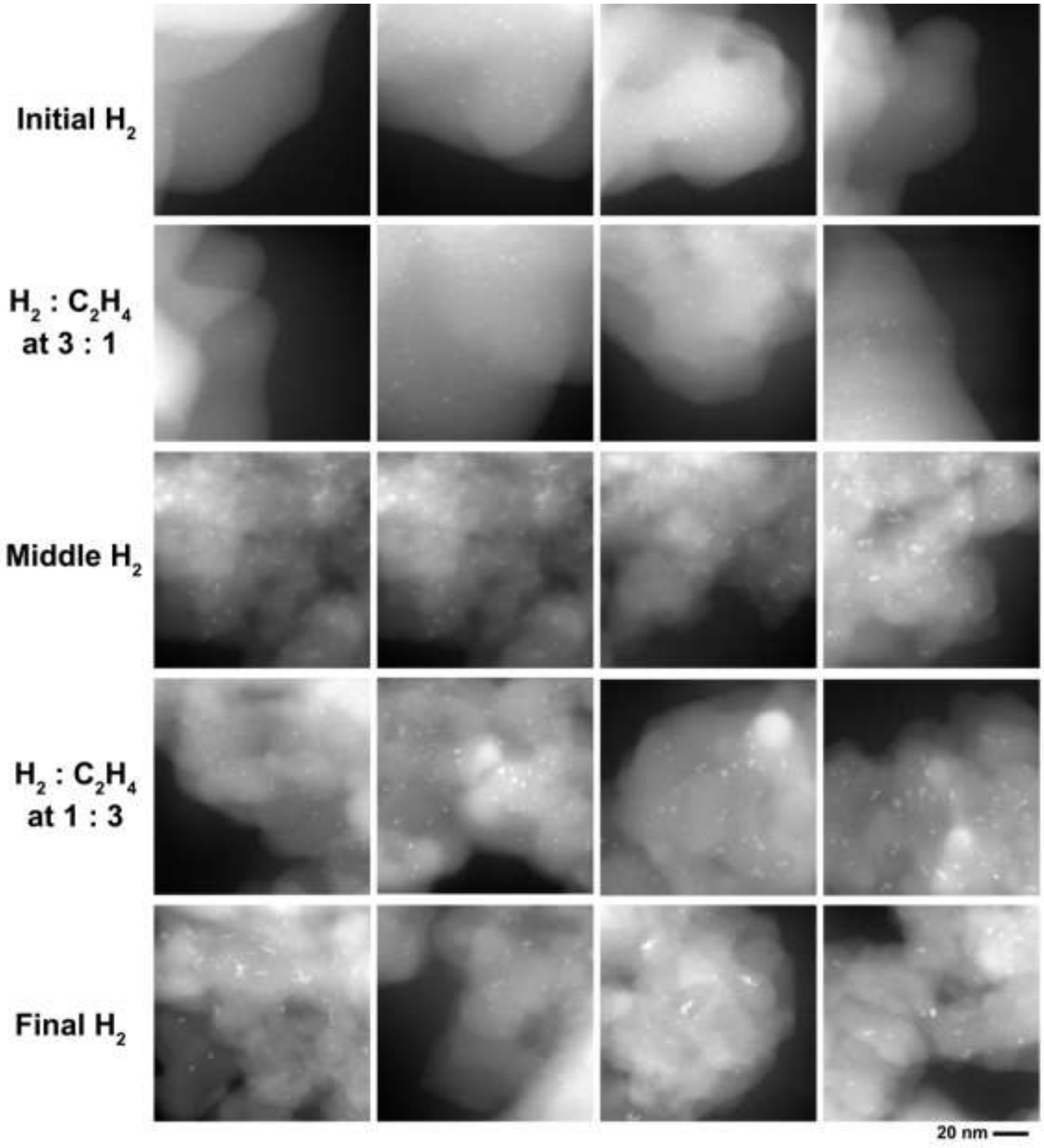

Supplementary Figure 3. Representative STEM images under five regimes: initial H_2, H_2 to C_2H_4 at 3:1, middle H_2, H_2 to C_2H_4 at 1:3 and final H_2.

3. Raw data and theoretical fits of XAFS spectra obtained in the two *operando* experiments: in the micro-cell and in a control experiment using Clausen cell.

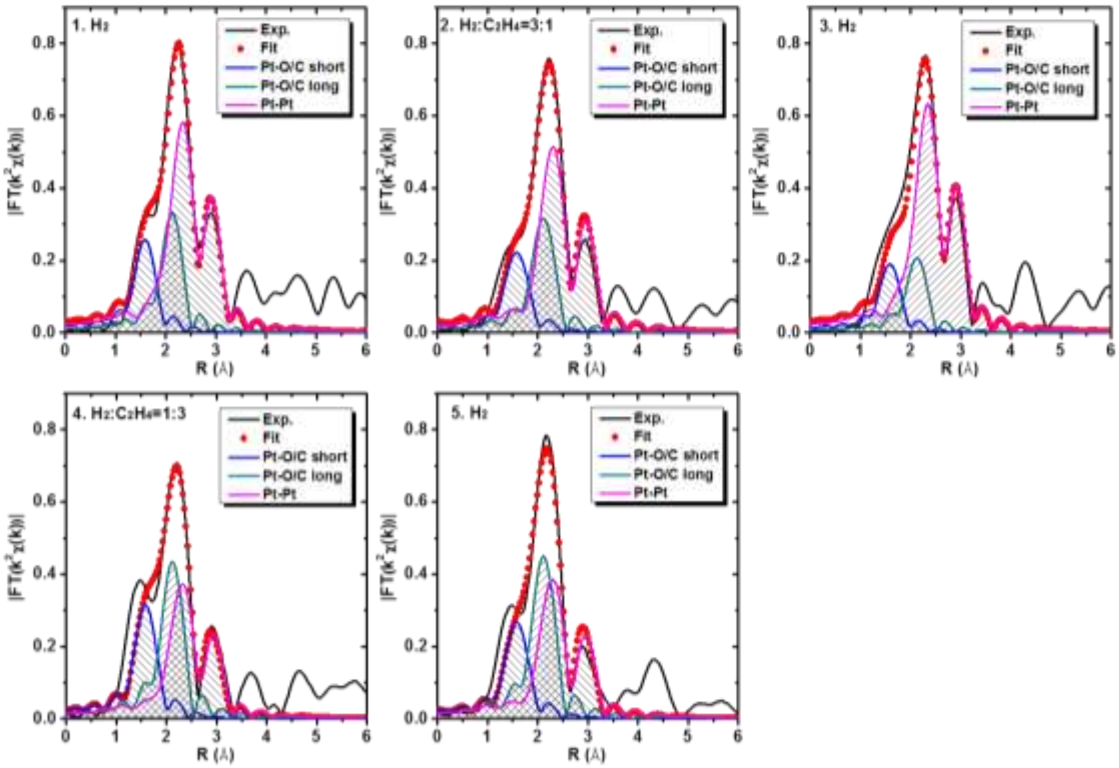

Supplementary Figure 4. The experimental and fit spectra for Pt/SiO_2 sample measured using micro-cell in five different regimes. The peaks filled with shadow indicate the contribution of Pt-O/C short bond (blue), Pt-O/C long bond and Pt-Pt bond to the fit.

In order to ensure that the changes in both the state of the catalyst and the gas composition were similar in the output of the microreactor and the Clausen cell[1], we performed a series of control experiments. The XAS data were collected in the same regimes twice. First, in the micro-reactor (at beamline X27A), and then, in a separate experiment – in the Clausen cell (at beamline X19A). At the X19A beamline, the sample was loaded into a quartz capillary (0.9 mm I. D. and 1.0 mm O.D.) and was treated in the same way in Clausen cell as in microcell, i.e. the gases were flowed follow the sequence of pure H_2, 3:1 of H_2: C_2H_4, pure H_2, 1:3 of H_2: C_2H_4, and pure H_2.

The XAFS data were collected in fluorescence mode that Clausen cell mounted on the stage was approximately 45 degrees to the incident X-ray beam and the emitted fluorescence was probed by PIPS detector. All XAFS spectra were recorded after the gases reached stable state. The behavior of the XAS data was qualitatively similar in the both experiments (Fig. 2 and Supplementary Fig. 5), validating our approach.

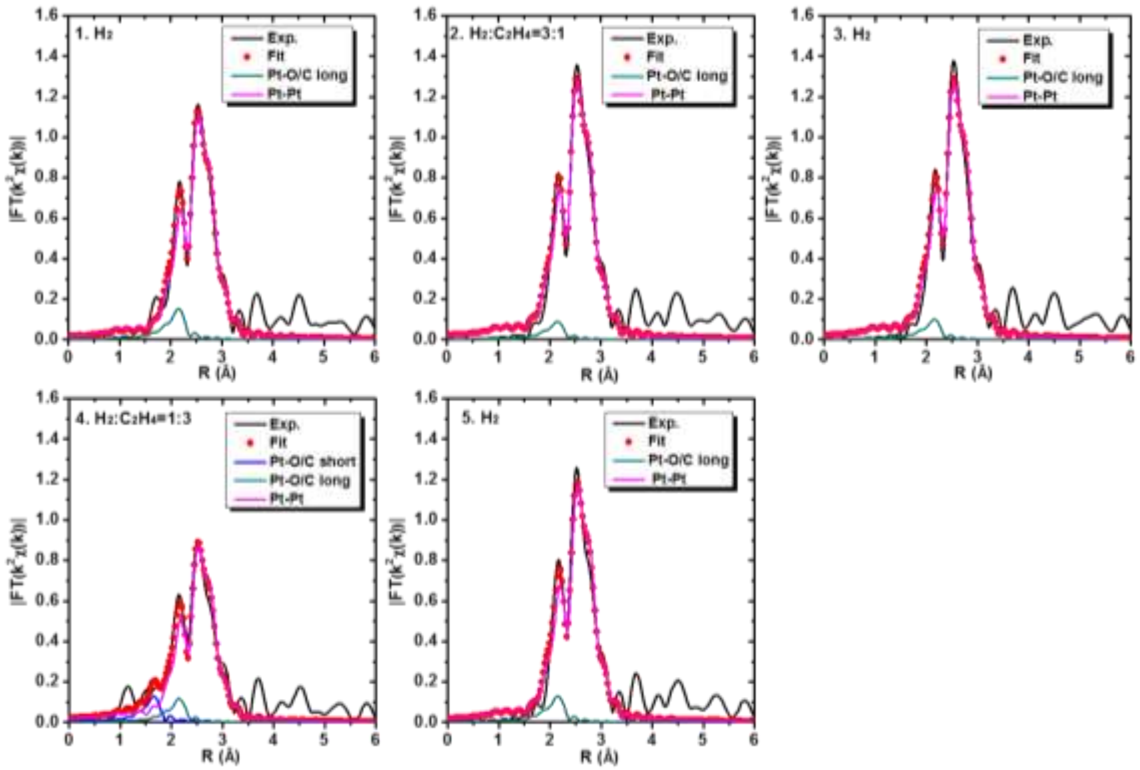

Supplementary Figure 5. The experimental and fit spectra for Pt/SiO_2 sample measured using Clausen cell in five different regimes. As shown in the figures, only for regime 4, all three paths were needed. For other regimes, two paths of Pt-O/C long and Pt-Pt were included for the fitting.

4. Representative STEM image of agglomerated Pt particles under H_2 flow.

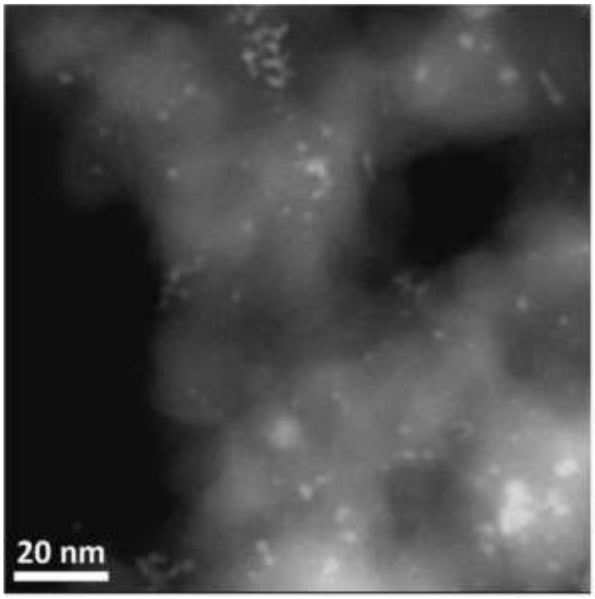

Supplementary Figure 6. An image of agglomerated particles in H_2 (regime 3).

5. Illustration of effects of carbon deposition under high image magnification.

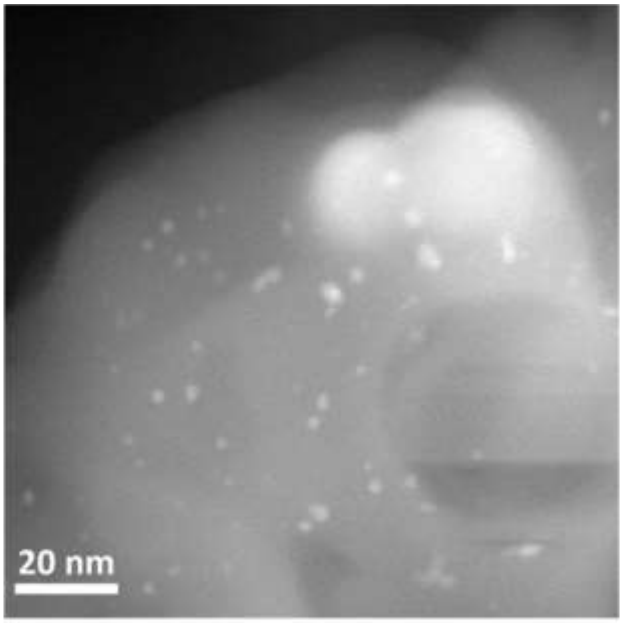

Supplementary Figure 7. Carbon existence/deposition as well as the beam damage in STEM experiment. Annular dark field scanning transmission electron microscopy images were acquired at a constant image magnification of 640,000.

6. Ex situ STEM results of Pt/SiO_2 catalyst before and after the reaction

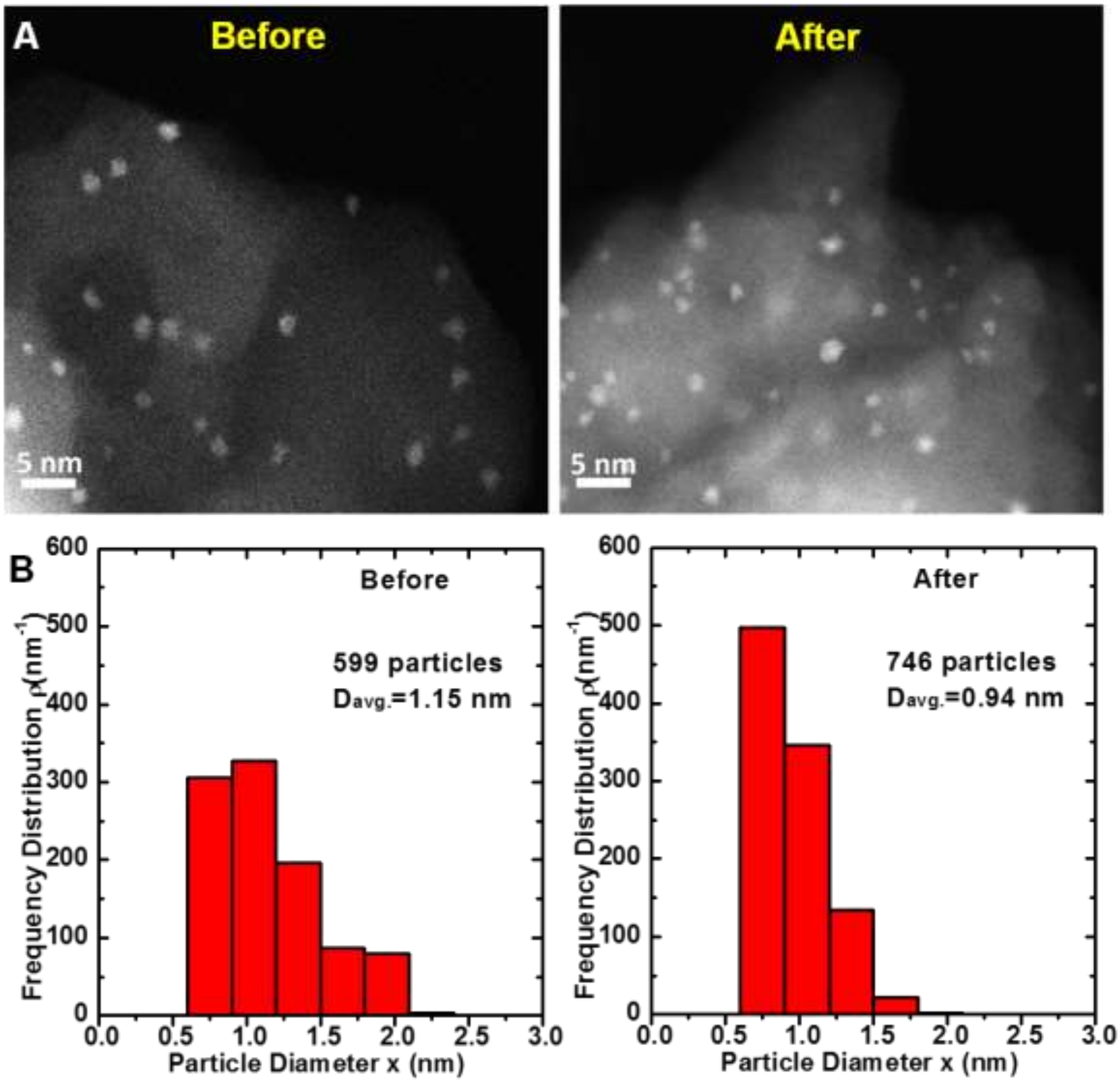

Supplementary Figure 8. ***Ex situ*** **STEM images (A) of Pt/SiO_2 catalyst before and after the reaction cycle. Histograms of size distribution, normalized by 1000 particles (B).**

Supplementary Tables

Table 1 | The summary of EXAFS fitting results for the data collected using micro-cell.

	Initial H_2	3:1 of H_2:C_2H_4	Middle H_2	1:3 of H_2:C_2H_4	Final H_2
N(Pt-Pt)	6.2±1.7	5.8±1.4	6.8±1.9	4.1±1.1	4.3±1.2
N(Pt-O/C short)	0.49±0.22	0.46±0.16	0.36±0.43	0.62±0.18	0.55±0.18
N(Pt-O/C long)	1.2±0.6	1.3±0.4	0.8±0.6	1.7±0.5	1.8±0.5
R(Pt-Pt) (Å)	2.74±0.01	2.74±0.01	2.74±0.01	2.74±0.01	2.74±0.01
R(Pt-O/C short) (Å)	1.99±0.02	1.99±0.02	1.99±0.02	1.99±0.02	1.99±0.01
R(Pt-O/C long) (Å)	2.55±0.02	2.55±0.02	2.55±0.01	2.55±0.01	2.55±0.01

ΔE_0=8.9±0.9 (eV), σ_i^2 for Pt-nonmetal paths is 0.000±0.004 $Å^2$, for Pt-Pt path is 0.008±0.002 $Å^2$.

Table 2 | The summary of EXAFS fitting results for the data collected using Clausen cell.

	Initial H_2	3:1 of H_2:C_2H_4	Middle H_2	1:3 of H_2:C_2H_4	Final H_2
N(Pt-Pt)	6.8±0.4	7.9±0.4	7.9±0.5	5.4±0.3	7.2±0.4
N(Pt-O/C short)	-	-	-	0.19±0.09	-
N(Pt-O/C long)	0.4±0.2	0.3±0.2	0.3±0.2	0.3±0.2	0.4±0.2
R(Pt-Pt) (Å)	2.746±0.003	2.743±0.002	2.745±0.003	2.737±0.003	2.746±0.002
R(Pt-O/C short) (Å)	-	-	-	2.00±0.03	-
R(Pt-O/C long) (Å)	2.51±0.02	2.51±0.02	2.51±0.02	2.51±0.02	2.51±0.02

ΔE_0=8.4±0.3 (eV), σ_i^2 for Pt-nonmetal paths is 0.000±0.002 $Å^2$, for Pt-Pt path is 0.0062±0.0002 $Å^2$.

Table 3 | The values of c, b and n_2 obtained according to Supplementary Equations 5-7 and particle size frequency distribution (Supplementary Fig. 2).

	Initial H_2	3:1 of H_2:C_2H_4	Middle H_2	1:3 of H_2:C_2H_4	Final H_2
$c=2m_2$	618911	390668	1227047	1719855	1252648
$b=N_2$	67605	45399	133966	186122	135548
$n_2 = 2m_2/N_2$	9.155	8.605	9.159	9.240	9.241

Table 4 | Numerical results for the number (N_1) of atoms in particles under the size resolution limit (1nm). The numbers were obtained according to Supplementary Equation 8 and numerical results of c, b and n_2 in Supplementary Table 3.

	N_1 Initial H_2	N_1 3:1 of H_2:C_2H_4	N_1 Middle H_2	N_1 1:3 of H_2:C_2H_4	N_1 Final H_2
Single atoms, Coordination number: n_1=0	32219	21958	46482	233355	155765
Dimers, n_1=1	38415	26532	54496	308631	202967
Trimers, n_1=2	47562	33514	65850	455598	291214
Tetrahedra, n_1=3	62425	45484	83178	869777	515224
Triangular bipyramid (5 atoms) n_1=3.6	76831	57888	98774	1913510	956845
Octahedra (6 atoms) n_1=4	90800	70752	112885	9567548	2232639
Truncated cuboctahedron (10 atoms), L=1, n_1=4.8	142686	127354	158039		

Table 5 | Number of particles above the size resolution limit (1nm) obtained by *operando* STEM.

	Initial H_2	3:1 of H_2:C_2H_4	Middle H_2	1:3 of H_2:C_2H_4	Final H_2
Number of particles above the size resolution limit (1nm)	361	400	666	812	612

Table 6 | Relative number of particles under the size resolution limit (1nm), normalized by the number of particles above the size resolution limit (Supplementary Table 5).

	Initial H_2	3:1 of H_2:C_2H_4	Initial H_2	1:3 of H_2:C_2H_4	Final H_2
Single atoms, Coordination number: n_1=0	89	55	70	287	255
Dimers, n_1=1	53	33	41	190	166
Trimers, n_1=2	44	28	33	187	159
Tetrahedra, n_1=3	43	28	31	268	210
Triangular bipyramid (5 atoms) n_1=3.6	43	29	30	471	313
Octahedra (6 atoms) n_1=4	42	29	28	1964	608
Truncated cuboctahedron (10 atoms), L=1, n_1=4.8	40	32	24	-	-

Table 7 | The numbers of agglomerates detected by *operando* STEM in five regimes.

	Initial H_2	3:1 of H_2:C_2H_4	Middle H_2	1:3 of H_2:C_2H_4	Final H_2
Number	0	0	12	3	12
Average size (nm)	0	0	2.40	2.74	2.22

Supplementary Discussion

Quantitative EXAFS analysis

EXAFS data were analyzed by using IFEFFIT (1.2.11c) package[2]. The program Athena (0.8.061)[3] was first used in order to process the raw data. The processing included the removal of smooth background from the measured absorption coefficient data, normalization of the X-ray absorption coefficient by the edge step and Fourier transform of the resultant spectrum from k-

space (k is photoelectron wave number) to r-space. Artemis (0.8.014c)[3] program was employed to fit the experimental data in r-space using theoretical EXAFS equation[4]:

$$\chi(k) = \sum_i \frac{S_0^2 N_i}{kR_i^2} | f_i^{eff}(k) | \sin[2kR_i - \frac{4}{3}\sigma_i^{(3)} k^3 + \delta_i(k)] e^{-2\sigma_i^2 k^2} e^{-2R/\lambda_i(k)} . \qquad (1)$$

In this equation, N_i is the number of atoms in the *i*th shell at the radius R_i around the central (absorbing) atom. S_0^2 is the passive electron reduction factor. It was fixed to be 0.862, the value obtained by fitting standard Pt foil. σ_i^2 is the mean squared disorder in the interatomic distances, known also as EXAFS Debye-Waller factor, $\sigma_i^{(3)}$ is the third cumulant, which was found to be consistent to zero, within the error bars, and not included in the final fits. The photoelectron mean free path, $\lambda_i(k)$, the photoelectron scattering-path amplitude, $f_i^{eff}(k)$, and phase, $\delta_i(k)$, were calculated with FEFF6[2] using model structures. In the fits, three single-scattering paths connecting Pt and its nearest neighbors were found to be the most important: 1) the Pt-O/C short bond (i=1, labeled as 1 in Fig. 2(b)), Pt-O/C long bond (i=2, labeled as 2 in Fig. 2(b)), and Pt-Pt bond (i=3, labeled as 3 in Fig. 2(b)). Because Pt-O and Pt-C paths have similar scattering amplitude and phase they cannot be distinguished by EXAFS analysis. Therefore, the Pt-nonmetal contribution is modeled as Pt-O.

To constrain fitting parameters and lower the uncertainties in the results, multiple data set fitting strategy was applied to all five experimental data. In this procedure, all experimental data were fit concurrently by their respective theoretical signals, while applying multiple constraints. Specifically, the photoelectron energy origin correction values (ΔE_0) were constrained to be the same for all paths. Short and long distances of Pt-O/C bonds were not allowed to vary between different regimes. The Debye Waller factors of all Pt-nonmetal bonds were constrained to be constant. To check the stability of this model, we compared its results against others in which

other fitting schemes were used (such as a single data set fit, or multiple data-set fits with fewer constraints), and the obtained trends were similar. The best fit results reported here were obtained using 45 independent points and 25 variables. The reduced Chi-square was obtained to be 3.87 and R-factor: 0.024. The fitting results for the data collected using micro-reactor were summarized in Supplementary Table 1 and the spectra were shown in Supplementary Fig. 4.

Compared to the data collected using micro-reactor (Supplementary Fig. 4), the spectra recorded using Clausen reactor (Supplementary Fig. 5) lacked a feature in the R range of 1-2 Å, except for the data in the regime 4 (mixture of H_2 and C_2H_4 with the ratio of 1:3). Therefore, in those fits the Pt-O/C short bond was not needed in four regimes. The fitting results were listed in Supplementary Table 2. The main trends in the behaviors of the Pt-Pt and Pt-nonmetal coordination numbers remain the same for the data measured using different reactors. Specifically, the coordination numbers of Pt-Pt decrease, and the coordination numbers of Pt-nonmetal bonds increase, in C_2H_4 rich condition (regime 4). This similarity validates our correlation between the XAFS and STEM results obtained in the micro-reactor and the reactivity data obtained in the downstream Clausen cell.

Determination of the number of particles in the size range below the STEM resolution limit (1 nm)

For a monatomic system, the coordination number of nearest neighbors to a "central" (e.g., X-ray absorbing) atom is defined as:

$$n = \frac{2m}{N}, \qquad (2)$$

where *m* is the total number of nearest neighbor (NN) pairs and *N* is the total number of atoms[5].

For an ensemble of monatomic particles that have a finite size distribution, coordination numbers obtained by EXAFS analysis will deviate from those that correspond to the particle of the average size[6]. The coordination number calculated for an ensemble of particles can be written as:

$$n = \frac{\int_0^\infty \rho(x) 2m(x)\, dx}{\int_0^\infty \rho(x) N(x)\, dx}, \tag{3}$$

where x is the cluster diameter, and $\rho(x) = dN/dx$ is the frequency distribution. While $\rho(x)$ measured by STEM can provide information about the relative number of particles of a certain size within the sample, there exists a cutoff, $x = a$, below which such information is unavailable. Simply put, there is an unknown number of particles whose size ranges between 0 and $x = a$ that cannot be accounted for by an *operando* STEM measurement. Our approach will rely on the measurement of the ensemble average coordination number, n, obtained by EXAFS, and the STEM-measured particle size distribution $\rho(x)$ with a known cutoff size, a.

We combine Supplementary Eqs. 2 and 3 to derive the relationship between the experimental observables, EXAFS-derived quantity on the left, and STEM-derived quantities on the right:

$$n = \frac{2m_a + \int_a^\infty \rho(x) 2m(x)\, dx}{N_a + \int_a^\infty \rho(x) N(x)\, dx}, \tag{4}$$

In our experiments the cutoff corresponds to a = 1nm for all distributions (only one is shown in Supplementary Fig. 2(A). Supplementary Eq. 4 enables an estimate of the unknown number of atoms, N_a and their interatomic pairs, m_a, which are "hidden" behind the size distribution cutoff.

Thus, our analysis method allows us to estimate the number of atoms unaccounted for by operando STEM.

The integrals on the right hand side of Supplementary Eq. 4 can be evaluated after approximating the larger clusters (with sizes larger than a) as regular polyhedra. This approximation works better for smaller clusters that are known to possess faceted shapes, while for larger clusters the detailed knowledge of morphology is not required because the surface to volume ratio is relatively small and the number of atoms $N(x)$ grows with size as, approximately, x^3, for all three-dimensional morphologies.

In this work, we prefer to adopt a particular morphology that was found in many supported metal clusters, specifically, a truncated *fcc* cuboctahedral model with the (111) cubic plane parallel to the support. Following Supplementary Ref.7, we will introduce a cluster order L, defined in regular polyhedral clusters as the number of spacing between adjacent atoms on the cluster edge. For cuboctahedral clusters, the cluster order L and cluster diameter x are simply related: $x = 2L\,r$, where r is the first nearest neighbor distance and the value of r is 2.75Å for nearest Pt-Pt. For example, for $L = 2$ the cluster base is the hexagon with diameter $x = 4r$.

In what will follow we will extend the formalism developed in Supplementary Ref.6 for regular polyhedral clusters to the hybrid case, where the known part of the size distribution (Supplementary Fig. 2) is approximated by a collection of regular truncated cuboctahedral clusters with orders L_i, and the unknown is described in terms of the number of atoms and bonds that are below the resolution cutoff. Therefore, Supplementary Eq. 4 will be rewritten as:

$$n = \frac{2m_1 + \sum_{L=L_{\min}} \rho(x_L) 2m(L)\,\Delta x}{N_1 + \sum_{L=L_{\min}} \rho(x_L) N(L)\,\Delta x}, \qquad (5)$$

where $\rho(x_L)$ is obtained from the original distribution $\rho(x)$ by interpolating it onto the $x_L = 2Lr$ grid, L is a cluster order that starts with the minimum order $L_{\min}$ corresponding to the cutoff distance, $x = a$, and $\Delta x = 2r$.

Calculations of $N(L)$ and $m(L)$ were obtained following the method described in Supplementary Ref.7:

$$N\left(L\right)=\frac{5}{3}L^3+4L^2+\frac{10}{3}L+1;\ \ 2m\left(L\right)=20L^3+21L^2+7L. \qquad (6)$$

Using Supplementary Eq. 5 we obtained N_1 and m_1, the total number of atoms and bonds, respectively, that contribute to the range of distances below the cutoff, a. Supplementary Equation 5 is rewritten as:

$$n=\frac{2m_1+c}{N_1+b}, \qquad (7)$$

We note that the meanings of c and b are the $2m_2$ and N_2, respectively, which correspond to the numbers of bonds and atoms in the measured part of the distribution. Accordingly, we introduce the $n_2 = 2m_2/N_2$ which is the coordination number of a “mean” cluster in the measured part of the distribution. Supplementary Table 3 contains the values of c, b and n_2 for different stages of the reaction.

Using Supplementary Equation 7, N_1 can be expressed as:

$$N_1=N_2\frac{n_2-n}{n-n_1}\ . \qquad (8)$$

In this equation, N_2 is the number of atoms in the measured part of the distribution and n_2 is the coordination number of a “mean” polyhedral cluster that corresponds to the entire measured

distribution. Though there are two variables in one equation, $2m_1$ and N_1, or n_1 and N_1, we can separately find the number of atoms N_1 below the resolution cutoff for each value of n_1, assuming for simplicity that only one type of clusters (with $n_1 = 0, 1, 2 < n$) is present there. The values of N_1 are listed in Supplementary Table 4. The data shown in Fig. 4 are obtained by normalizing the calculated values of N_1 (Supplementary Eq. 8 and Supplementary Table 4) for all types of Pt species by the number of particles in the visible part of the distribution in each regime (shown in Supplementary Table 5). The data shown in Fig. 4 are tabulated in Supplementary Table 6. In Supplementary Table 6, the number of particles does not include the contribution of agglomerated particles (shown separately in Supplementary Table 7), as the STEM data indicate that they do not contribute a significant additional mass to affect the analysis.

Supplementary References

1. Clausen, B. S. *et al.* In situ cell for combined XRD and on-line catalysis tests: studies of Cu-based water gas shift and methanol catalysts. *J. Catal.* **132**, 524-535 (1991).
2. Newville, M. IFEFFIT : interactive XAFS analysis and FEFF fitting. *J. Synchrotron Rad.* **8**, 322-324 (2001).
3. Ravel, B. & Newville, M. ATHENA, ARTEMIS, HEPHAESTUS: data analysis for X-ray absorption spectroscopy using IFEFFIT. *J. Synchrotron Rad.* **12**, 537-541 (2005).
4. Zabinsky, S. I., Rehr, J. J., Ankudinov, A., Albers, R. C. & Eller, M. J. Multiple-scattering calculations of x-ray-absorption spectra. *Phys. Rev. B* **52**, 2995-3009 (1995).
5. Frenkel, A. I. Applications of extended X-ray absorption fine-structure spectroscopy to studies of bimetallic nanoparticle catalysts. *Chem. Soc. Rev.* **41**, 8163-8178 (2012).
6. Frenkel, A. I., Yevick, A., Cooper, C. & Vasic, R. Modeling the structure and composition of nanoparticles by extended X-ray absorption fine structure spectroscopy. *Annu. Rev. Anal. Chem.* **4**, 23-39 (2011).
7. MontejanoCarrizales, J. M., AguileraGranja, F. & MoranLopez, J. L. Direct enumeration of the geometrical characteristics of clusters. *Nanostruct. Mater.* **8**, 269-287 (1997).
